# Patent Medicines

**Published:** 1858-07

**Authors:** 


### PATENT MEDICINES.

THE editor of the "Letter Box" says: that within a year, he "has taken pains to count the different medicinal preparations offered for sale for the cure of human ailments, and that they number over FIFTEEN HUNDRED; and that among all that are liquid, there is not one which does not contain either opium or alcohol." Still, newspapers, secular and religious, advertise these without compunction, when they would be horrified to see in their columns, even by mistake, an advertisement to sell "pure liquors" by the glass or barrel. Perhaps conscience is quieted in this way: "Pure liquors are certainly mischievous; but if they are rendered impure, by putting medicine into them, they may do some good." But yet these hair-splitting gentlemen launch out their severest anathemas against the "unprincipled men who fabricate wines, and brandies, beers and other forms of alcohol." Every liquid patent medicine is nothing more or less than disguised alcohol or opium. Fanatical men exclude wine from the Communion Table; so tee-total are they, that a drop is not admissible under any ordinary circumstances; and yet they advertise, and purchase, and swallow, and commend what is essentially alcohol—only it is *called* medicine, somebody's "bitters" or "tonic." If alcohol is essentially pernicious—poisonous—as is claimed by Temperance men, it is not the less so for being simply disguised by some other name or ingredient. If we hope for victory, we must be consistent; and it is naturally considered that consistency—a firm adherence to solid principles—should commence with the religious press, and with the respectable secular newspapers, and let Tonics, Bitters, Renovators, Schnapps, Made Brandy, Beastly Beers, and Rot-Gut Whisky, be considered as in the same category.

